# Hallmarks of NLRP3 inflammasome activation are observed in organotypic hippocampal slice culture

**DOI:** 10.1111/imm.13221

**Published:** 2020-06-22

**Authors:** Christopher Hoyle, Elena Redondo‐Castro, James Cook, Te‐Chen Tzeng, Stuart M. Allan, David Brough, Eloise Lemarchand

**Affiliations:** ^1^ Division of Neuroscience and Experimental Psychology School of Biological Sciences Faculty of Biology, Medicine and Health Manchester Academic Health Science Centre University of Manchester Manchester UK; ^2^ The Lydia Becker Institute of Immunology and Inflammation University of Manchester Manchester UK; ^3^ Immunology and Inflammation Bristol‐Myers Squibb (Celgene Corporation) Cambridge MA USA

**Keywords:** interleukin‐1*β*, inflammasome, microglia, NLRP3, organotypic hippocampal slice culture

## Abstract

Microglial inflammation driven by the NACHT, LRR and PYD domain‐containing protein 3 (NLRP3) inflammasome contributes to brain disease and is a therapeutic target. Most mechanistic studies on NLRP3 activation use two‐dimensional pure microglial cell culture systems. Here we studied the activation of the NLRP3 inflammasome in organotypic hippocampal slices, which allowed us to investigate microglial NLRP3 activation in a three‐dimensional, complex tissue architecture. Toll‐like receptor 2 and 4 activation primed microglial inflammasome responses in hippocampal slices by increasing NLRP3 and interleukin‐1*β* expression. Nigericin‐induced NLRP3 inflammasome activation was dynamically visualized in microglia through ASC speck formation. Downstream caspase‐1 activation, gasdermin D cleavage, pyroptotic cell death and interleukin‐1*β* release were also detected, and these findings were consistent when using different NLRP3 stimuli such as ATP and imiquimod. NLRP3 inflammasome pathway inhibitors were effective in organotypic hippocampal slices. Hence, we have highlighted organotypic hippocampal slice culture as a valuable *ex vivo* tool to allow the future study of NLRP3 inflammasomes in a representative tissue section, aiding the discovery of further mechanistic insights and drug development.

## Graphical abstract

We used organotypic hippocampal slices to investigate microglial NLRP3 inflammasome responses in a three‐dimensional, complex environment. Priming of NLRP3 inflammasome responses was observed in microglia, and ASC speck formation was dynamically visualized in hippocampal slices. Activation of NLRP3 in microglia in hippocampal slices led to caspase‐1 activation, gasdermin D cleavage and pyroptosis.

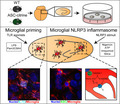

**Abbreviations**
3D: three‐dimensional
ASC: apoptosis‐associated speck‐like protein containing a caspase recruitment domain
ATP: adenosine triphosphate
BSA: bovine serum albumin
ELISA: enzyme‐linked immunosorbent assay
FITC: fluorescein isothiocyanate
IB4: isolectin GS‐IB4 AlexaFluor™ 594 conjugate
IL: interleukin
LPS: lipopolysaccharide
NLRP3: NACHT, LRR and PYD domain‐containing protein 3
OHSC: organotypic hippocampal slice culture
PBS: phosphate‐buffered saline
PBST: phosphate‐buffered saline, 0·1% Tween‐20
PCR: polymerase chain reaction
PenStrep: penicillin and streptomycin
PI: propidium iodide
SEM: standard error of the mean
TLR: Toll‐like receptor
TNF: tumour necrosis factor
WT: wild‐type

## Introduction

Microglia are highly dynamic macrophage‐like cells that play an integral role in brain development and maintain brain tissue homeostasis by surveillance of the local microenvironment.^1^ However, microglia also have a critical role in brain pathology, contributing to the progression of diseases such as Alzheimer’s disease, Parkinson’s disease and stroke through the generation of a neuroinflammatory response within the brain parenchyma.^2^ To reveal new therapeutic targets for brain disease, it is important to identify the cellular mechanisms underlying damaging neuroinflammation. Inflammasomes are important innate immune complexes that regulate the activation of the protease caspase‐1 and subsequent maturation and release of the pro‐inflammatory cytokines interleukin‐1*β* (IL‐1*β*) and IL‐18.^3^ Several inflammasomes have been described, of which the NACHT, LRR and PYD domain‐containing protein 3 (NLRP3) inflammasome is considered important for driving inflammatory responses in the absence of infection.^4^ Without an obvious causal role for infection, the inflammation worsening many brain pathologies is probably caused by host‐derived damage‐associated stimuli. Hence, the NLRP3 inflammasome is often implicated in the pathology of brain diseases.^5^

Canonical NLRP3 inflammasome activation requires two signals. Signal one is a priming stimulus that typically activates Toll‐like receptor (TLR4) or TLR2, driving nuclear factor‐*κ*B‐dependent expression of inflammatory proteins including NLRP3 and pro‐IL‐1*β*, as well as post‐translational licensing of NLRP3 through de‐ubiquitination, phosphorylation and de‐sumoylation.^6^, ^7^, ^8^, ^9^ Macrophage metabolic reprogramming upon TLR4 activation also regulates the priming response.^10^, ^11^ Following priming, a second pathogen‐associated molecular pattern or damage‐associated molecular pattern signal activates NLRP3. Examples of the second NLRP3 activating stimulus include the bacterial toxin nigericin, extracellular adenosine triphosphate (ATP), aggregated amyloid‐*β* and silica crystals.^12^, ^13^, ^14^, ^15^ A common feature of the secondary stimuli is often potassium ion efflux from the cell, which is established as a trigger for formation of the NLRP3 inflammasome complex.^16^ Potassium ion efflux‐independent NLRP3 activation can also be induced by the TLR7 agonist imiquimod.^17^ Activated NLRP3 interacts with apoptosis‐associated speck‐like protein containing a caspase recruitment domain (ASC), which polymerizes in the cytosol to form an ASC speck.^18^, ^19^ ASC speck formation facilitates the recruitment and activation of the inflammasome effector protein caspase‐1, which in turn induces the proteolytic maturation of pro‐IL‐1*β*, allowing mature IL‐1*β* release from the cell.^20^ Caspase‐1 also triggers cleavage of gasdermin D, the N‐terminus of which subsequently forms membrane pores leading to pyroptotic cell death, and which may also form a conduit for IL‐1*β* release.^21^, ^22^, ^23^, ^24^ Caspase‐1 autoprocessing subsequently terminates its enzymatic activity, limiting the inflammatory response.^25^

Several reports describe canonical NLRP3 inflammasome activation in microglial cultures.^12^, ^26^, ^27^, ^28^ However, few studies have demonstrated NLRP3 inflammasome formation in the brain parenchyma. Bernardino *et al*.^29^ reported IL‐1*β* release from murine organotypic hippocampal slice cultures in response to lipopolysaccharide (LPS) and ATP stimulation, and the same treatment also induced IL‐1*β* release from acutely isolated murine and rat hippocampal slices.^30^ The presence of microglial and extracellular NLRP3 inflammasomes has been demonstrated both in the post‐mortem brains of people with Alzheimer’s disease and in APP/PS1 transgenic mice, as well as in the brains of mice undergoing experimental cerebral malaria.^31^, ^32^, ^33^

Despite compelling evidence to suggest an important role for microglial NLRP3 inflammasome responses in brain pathology, our understanding of these pathways within the brain parenchyma remains limited. This is, in part, a result of a lack of *ex vivo* tools to interrogate NLRP3 inflammasome pathways in a complex brain‐like environment. Much of our knowledge is derived from *in vitro* studies using microglial cell lines or primary microglial cultures, which exhibit an altered transcriptomic profile compared with freshly isolated adult microglia, and this is compounded by the absence of other brain cell types.^34^ There is a clear need for a tractable, robust microglial model that better reflects the *in vivo* setting, facilitating a greater understanding of the underlying neuroinflammatory biology. Here, we used organotypic hippocampal slice cultures (OHSCs),^35^ in which the three‐dimensional (3D) cellular architecture of the hippocampus was maintained, to investigate microglial NLRP3 inflammasome responses. We demonstrate both priming and activation of NLRP3 inflammasome responses in OHSC microglia. ASC speck formation, caspase‐1 activation and cleavage of gasdermin D and IL‐1*β* were detected in OHSCs, and inhibitors of the NLRP3 inflammasome pathway showed efficacy in this model. Hence, we have established OHSCs as a valuable *ex vivo* tool to interrogate microglial NLRP3 inflammasome responses.

## Materials and methods

### Mice

Experiments were carried out on an in‐house colony of wild‐type (WT) or ASC–citrine^36^ C57BL/6 mice at the University of Manchester. Animals were allowed free access to food and water and maintained under temperature‐, humidity‐ and light‐controlled conditions. All animal procedures adhered to the UK Animals (Scientific Procedures) Act (1986).

### OHSC preparation

Seven‐day‐old mouse pups of either sex were killed by cervical dislocation and the brains were collected in phosphate‐buffered saline (PBS) containing glucose (5 mg/ml). Hippocampi were dissected, placed on filter paper and 400‐μm slices were cut using a McIlwain tissue chopper (Brinkman Instruments, Runcorn, UK). Hippocampal slices were collected and placed on 0·4‐μm Millicell culture inserts (Millipore, Watford, UK), as previously described by Stoppini *et al*.^37^ Three hippocampal slices were placed on each insert. Slices were maintained in a humidified incubator with 5% CO_2_ at 37° with 1 ml of minimal essential medium (Gibco, Waltham, MA) containing 20% horse serum (Sigma, Poole, UK), supplemented with HEPES (30 mm; ThermoFisher Scientific, Waltham, MA) and insulin (0·1 mg/ml; Gibco), pH 7·2–7·3. Culture medium was changed every 2 days, and slices were used at day 7.

### Primary murine adult microglia preparation

Mice aged 8–10 weeks of either sex were deeply anaesthetized with isofluorane and transcardially perfused with ice‐cold PBS. Unless otherwise stated, all subsequent steps were performed on ice or at 4°. Brains (excluding cerebellum) were excised, minced with a disposable scalpel into 1‐ to 3‐mm^3^ chunks, and digested using a Neural Tissue Dissociation Kit (Miltenyi, Surrey, UK) according to the manufacturer’s instructions. The resulting suspension was homogenized in a Dounce tissue grinder (Sigma) with 20 strokes of a loose‐clearance pestle. The resulting single‐cell suspension was depleted of myelin by centrifuging in 33% Percoll (GE Healthcare, Amersham, UK) for 10 min at 1000 *g* (low brake) and aspirating the myelin layer. Cells were then pelleted by diluting 1 : 4 in Hanks’ balanced salt solution without calcium or magnesium (Gibco) and centrifuging for 10 min at 500 *g*. Microglia were magnetically labelled with 10 μl anti‐CD11b magnetic microbeads (Miltenyi) per brain in MACS buffer [PBS without calcium or magnesium, with 2 mm ethylenediaminetetraacetic acid and 0·5% bovine serum albumin (BSA)] for 15 min at 4° followed by positive enrichment on LS columns (Miltenyi) according to the manufacturer’s instructions. The resulting microglia suspension was pelleted, counted and spot‐plated onto 96‐well Cell+ plates (Sarstedt, Nuembrecht, Germany) at a density of 10 000 cells per well. The plates were incubated briefly at 37° to allow cell attachment, following which the wells were replenished with 100 μl of culture medium [Dulbecco’s modified Eagle’s medium/F12 (Sigma) containing 10% fetal bovine serum (ThermoFisher), 100 U/ml of penicillin and 100 µg/ml streptomycin (PenStrep; ThermoFisher), 2 mm glutamine) supplemented with IL‐34 (20 ng/ml; R&D Systems, Abingdon, UK) and transforming growth factor‐*β*_1_ (50 ng/ml; Miltenyi)]. Cells were used at day 7.

### Primary murine mixed glial culture preparation

Murine mixed glial cells were prepared from the brains of 2‐ to 4‐day‐old mice of either sex that were killed by cervical dislocation. The brains were isolated and the cerebral hemispheres were dissected and the meninges were removed. The remaining tissue was homogenized in Dulbecco’s modified Eagle’s medium containing 10% fetal bovine serum and PenStrep via repeated trituration. The resulting homogenate was centrifuged at 500 *g* for 10 min and the pellet was resuspended in fresh culture medium before being incubated in a flask at 37°, 90% humidity and 5% CO_2_. After 5 days, the cells were washed and fresh medium was placed on the cells. The medium was replaced every 2 days. On day 12, the cells were seeded at 2 × 10^5 ^cells/ml in 24‐well plates and incubated for a further 2 days before use.

### Priming assays

The OHSCs, adult microglia and murine mixed glia were primed with vehicle (PBS), LPS (1 µg/ml; Sigma) or Pam3CSK4 (100 ng/ml, 3 hr; InvivoGen, Toulouse, France) in culture medium containing serum. Supernatants were collected, and OHSCs were prepared for quantitative polymerase chain reaction (PCR), immunofluorescence staining or Western blotting.

### OHSC inflammasome activation assays

The OHSCs were primed with LPS (1 µg/ml, 3 hr) in culture medium containing serum. The medium was then replaced with serum‐free culture medium, and in some experiments, drugs were spiked into this medium for 15–60 min. These drugs were MCC950 (10 µm; Sigma), ac‐YVAD‐cmk (100 µm; VWR International, Lutterworth, UK), VX‐765 (10 µm; Selleckchem), ZVAD‐fmk (50 µm; Merck, Watford, UK) and NBC19 (25 µm). After drug pre‐treatment, the following soluble NLRP3 inflammasome stimuli were spiked into the culture medium: nigericin (10 µm, 0–90 min; Sigma), ATP (5 mm, 90 min; Sigma) and imiquimod (75 µm, 2 hr; InvivoGen). To activate the NLRP3 inflammasome with insoluble stimuli, silica (3 µl, 3 mg/ml, 24 hr; US Silica, Katy, TX) was pipetted directly on top of each hippocampal slice on each insert. Following inflammasome activation, culture medium was collected and analysed for cytokine content by enzyme‐linked immunosorbent assay (ELISA), or for content of specific proteins by Western blotting. Hippocampal slices were prepared for either Western blotting or immunostaining.

### Adult microglia and mixed glia inflammasome activation assays

Cells were primed with LPS (1 µg/ml, 3 hr) in culture medium containing serum. The medium was then replaced with serum‐free culture medium in the presence or absence of MCC950 (10 µm, 15 min). After drug pre‐treatment, NLRP3 inflammasome activation was induced by in the addition of nigericin (10 µm, 60 min), ATP (5 mm, 60 min) or imiquimod (75 µm, 2 hr). Silica (0·3 mg/ml) was added simultaneously with MCC950 for 24 hr (adult microglia) or 4 hr (mixed glia).

### Western blotting

The OHSCs, adult microglia and mixed glia were lysed with lysis buffer (50 mm Tris–HCl, 150 mm NaCl, Triton 1% volume/volume, pH 7·3) containing protease inhibitor cocktail (Calbiochem, Watford, UK). The OHSCs were additionally lysed using repeated trituration and brief water bath sonication. Lysates were then centrifuged for 10 min at 12 000 *g* at 4° and analysed for pro‐IL‐1*β*, mature IL‐1*β*, NLRP3, pro‐caspase‐1, caspase‐1 p10 and gasdermin D. Equal amounts of protein were loaded from lysates, whereas equal volumes of supernatants were loaded. Samples were run on sodium dodecyl sul[hate–polyacrylamide gels and transferred at 25 V onto nitrocellulose or PVDF membranes using a Trans‐Blot^®^ Turbo Transfer™ System (Bio‐Rad, Watford, UK). Membranes were blocked in either 5% weight/volume milk or 2·5% BSA (Sigma) in PBS, 0·1% Tween‐20 (PBST) for 1 hr at room temperature. The membranes were then washed with PBST and incubated at 4° overnight with goat anti‐mouse IL‐1*β* (250 ng/ml; R&D Systems, Cat# AF‐401‐NA), mouse anti‐mouse NLRP3 (1 µg/ml; Adipogen, Cat# AG‐20B‐0014‐C100), rabbit anti‐mouse caspase‐1 (1·87 µg/ml; Abcam, Waltham, MA, Cat# ab179515) or rabbit anti‐mouse gasdermin D (0·62 µg/ml; Abcam, Cat# ab209845) primary antibodies in 0·1% (IL‐1*β*), 1% (NLRP3) or 2·5% (caspase‐1, gasdermin D) BSA in PBST. Membranes were washed and incubated with rabbit anti‐goat (500 ng/ml, 5% milk in PBST; Dako, Stockport, UK, Cat# P044901‐2), rabbit anti‐mouse (1·3 µg/ml, 5% milk in PBST; Dako, Cat# P026002‐2) or goat anti‐rabbit IgG (250 ng/ml, 2·5% BSA in PBST; Dako, Cat# P044801‐2) at room temperature for 1 hr. Proteins were then visualized with Amersham ECL Western Blotting Detection Reagent (GE Life Sciences, Amersham, UK) and G:BOX (Syngene, Cambridge, UK) and Genesys software (Syngene). *β*‐Actin (Sigma, Cat# A3854) was used as a loading control.

### Immunostaining

The OHSCs were washed once with cold PBS and fixed in 4% paraformaldehyde (1 hr) at 4°. OHSCs were washed twice more in cold PBS and then incubated with goat anti‐mouse IL‐1*β* (2 µg/ml; R&D Systems, Cat# AF‐401‐NA), rabbit anti‐mouse Iba‐1 (1·294 µg/ml; Abcam, Cat# ab178846) or rabbit anti‐mouse ASC (202 ng/ml; Cell Signaling Technology, Waltham, MA, Cat# 67824) primary antibodies overnight at 4°. OHSCs were washed and incubated with AlexaFluor™ 488 donkey anti‐rabbit IgG (2 µg/ml; Invitrogen, Waltham, MA, Cat# A‐21206), AlexaFluor 594 donkey anti‐rabbit IgG (2 µg/ml; Invitrogen, Cat# A‐21207) or AlexaFluor 647 donkey anti‐rabbit IgG (4 µg/ml; Invitrogen, Cat# A32795) secondary antibodies for 2 hr at room temperature. For IL‐1*β* immunostaining, biotin amplification was performed using biotinylated anti‐goat IgG antibody (7·5 µg/ml, 2 hr at room temperature; Vector Laboratories, Peterborough, UK, Cat# BA‐9500), before washing and subsequent incubation in streptavidin AlexaFluor 546 conjugate (5 µg/ml; Invitrogen, Cat# S11225). All antibody incubations were performed using PBS, 0·3% Triton X‐100. Wash steps were performed using PBST unless stated otherwise. OHSCs were washed and then incubated in DAPI (1 µg/ml, 15 min; Sigma, Cat# D9542) at room temperature before final washing and mounting using ProLong gold antifade mountant (ThermoFisher) before imaging using wide‐field or confocal microscopy.

### Reverse transcription quantitative PCR

Total RNA was extracted from samples with TRIzol Reagent (ThermoFisher) according to the manufacturer’s instructions. RNA (1 µg) was converted to cDNA using Superscript III Reverse Transcriptase (ThermoFisher). Quantitative PCR was performed using Power SYBR® Green PCR Master Mix (ThermoFisher) in a 384‐well format using a 7900HT Fast Real‐Time PCR System (Applied Biosystems, Waltham, MA), and 3 μl of 1 : 20‐diluted cDNA was loaded with 200 nmol/l of primers in triplicate. Data were normalized to the expression of the housekeeping gene *Hmbs* or *Actb*. Expression levels of genes of interest were computed as follows: relative mRNA expression = *E* ^−(^ *^C^* ^t of gene of interest)^/ *E* ^−(^ *^C^* ^t of housekeeping gene)^, where *C*_t_ is the threshold cycle value and *E* is efficiency. Primers used were: NLRP3 forward, GCCCAAGGAGGAAGAAGAAG, NLRP3 reverse, TCCGGTTGGTGCTTAGACTT; Pycard forward, TGCTTAGAGACATGGGCTTACA, Pycard reverse, ACTCTGAGCAGGGACACTGG; Caspase‐1 forward, CATTTGTAATGAAGACTGCTACCTG, Capase‐1 reverse, GATGTCCTCCTTTAGAATCTTCTGT; GSDMD forward, TGCAGATCACTGAGGTCCAC, GSDMD reverse, GCCTTCACCCTTCAAGCATA; IL‐1*β* forward, AACCTGCTGGTGTGTGACGTTC, IL‐1*β* reverse, CAGCACGAGGCTTTTTTGTTGT; IL‐18 forward, GACTCTTGCGTCAACTTCAAGG, IL‐18 reverse, CAGGCTGTCTTTTGTCAACGA; tumour necrosis factor (TNF) forward, CCCTCACACTCAGATCATCTTCT, TNF reverse, GCTACGACGTGGGCTACAG; IL‐1*α* forward, TCTCAGATTCACAACTGTTCGTG, IL‐1*α* reverse, AGAAAATGAGGTCGGTCTCACTA; IL‐6 forward, CTCTGGGAAATCGTGGAAAT, IL‐6 reverse, CCAGTTTGGTAGCATCCATC.

### ELISA

The levels of IL‐1*β* in the supernatant were analysed by ELISA (DuoSet, R&D Systems) according to the manufacturer’s instructions.

### Cell death assays

Cell death in the hippocampal slices was assessed by adding propidium iodide (25 µg/ml; Sigma) to the culture medium for the final 30 min of inflammasome activation. Cell death in microglial cultures was assessed by measuring lactate dehydrogenase release into the supernatant using a CytoTox 96 Non‐Radioactive Cytotoxicity Assay (Promega, Southampton, UK) according to the manufacturer’s instructions. Supernatants containing silica were centrifuged at 12 000 *g* (10 min) at 4° before analysis to remove silica.

### Snapshot wide‐field microscopy

Images were collected on a Zeiss Axioimager.M2 upright microscope using a 5× or 20× Plan Apochromat objective and captured using a Coolsnap HQ2 camera (Photometrics, Birmingham, UK) through micromanager software (v1.4.23). Specific band‐pass filter sets for DAPI, fluorescein isothiocyanate (FITC), Cy3 and Texas Red were used to prevent bleed‐through from one channel to the next.

### Confocal microscopy

Images were collected on a Leica TCS SP8 AOBS upright confocal microscope through LAS X (v3.5.2.18963) using a 63X/1.40 HCS PL Apo objective and 1× confocal zoom. The confocal settings were as follows: pinhole 1 airy unit, scan speed 400 Hz bidirectional, format 1024 × 1024. The white‐light laser was used with FITC 488 nm, Cy3 555 nm, Texas Red 594 nm and Cy5 647 nm laser lines. Images were collected using hybrid and photon‐multiplying tube detectors with the following detection mirror settings: DAPI 410–520 nm, Cy3 565–636 nm and Cy5 657–744 nm for IL‐1*β*/Iba1 images; and DAPI 410–478 nm, FITC 498–584 nm and Texas Red 604–749 nm for ASC/Iba1 images. Images were collected sequentially to eliminate crosstalk between channels. For live imaging, OHSCs were LPS‐primed (1 µg/ml, 3 hr) and then medium was replaced with phenol‐red‐free, serum‐free culture medium containing Hoechst (2 µg/ml; ThermoFisher) and isolectin GS‐IB4–AlexaFluor 594 conjugate (from *Griffonia simplicifolia*; IB4; 5 µg/ml; ThermoFisher) and incubated for 2 hr at 37°. OHSCs were subsequently covered with 1·5 ml phenol‐red‐free, serum‐free medium to allow lens immersion, and then nigericin (10 µm) was spiked into the culture medium underneath the insert. The OHSCs were then placed into a confocal microscope chamber heated to 37° and imaged for 60–90 min.

### Image processing analysis

Analysis was performed using FIJI (imagej, NIH, Bethesda, MD) on images acquired from the same region of three separate OHSCs (from the same insert) per treatment, and these values were averaged for each biological repeat. IL‐1*β* and Iba1 colocalization was quantified on 63× z‐stacks acquired on a confocal microscope. The number of IL‐1*β*‐positive cells in each stack was counted manually. Each of these IL‐1*β*‐positive cells was simultaneously assessed for Iba1 signal. ASC speck formation was quantified on 20× wide‐field microscopy images by subtracting background (50‐pixel rolling ball radius), autothresholding using the triangle method and analysing particles with the following thresholds: size 1–10 μm^2^, circularity 0·9–1·0. In cases of low or no ASC speck formation, manual thresholding was performed to ensure accurate quantification of ASC speck number. To quantify propidium iodide (PI) uptake, images were acquired on a wide‐field microscope using a 5× objective, background was subtracted (5.0‐pixel rolling ball radius) and images were automatically thresholded using the default method. In cases of low or no detectable cell death, images were manually thresholded to ensure that all PI‐positive cells were included. The total area of PI‐positive signal was measured in the whole field of view, and was then normalized to the total area of DAPI signal. ASC speck spatial localization was determined using 63× z‐stacks acquired on a confocal microscope. The total number of ASC specks through the z‐stack was counted manually, and each speck present in an Iba1‐positive cell was classed as perinuclear (≤ 2 μm from the nucleus) or cytosolic (> 2 μm from the nucleus). ASC specks that were not in an Iba1‐positive cell were labelled as unclassified. All 3D reconstructions from z‐stacks were generated using imaris software (v9.3.1; Bitplane, Zurich, Switzerland).

### Statistical analysis

Data are presented as the mean ± standard error of the mean (SEM) overlaid with individual data points. Transformations or corrections were applied as necessary to obtain equal variance between groups before analysis. Data were analysed using two‐tailed unpaired *t*‐tests or repeated‐measures one‐way analysis of variance with Dunnett’s *post‐hoc* test using graphpad prism (v7; GraphPad, San Diego, CA). Statistical significance was accepted at *P < 0·05, **P < 0·01 and ***P < 0·001.

## Results

### NLRP3 inflammasome priming by TLR stimuli in OHSCs

**Figure 1:**
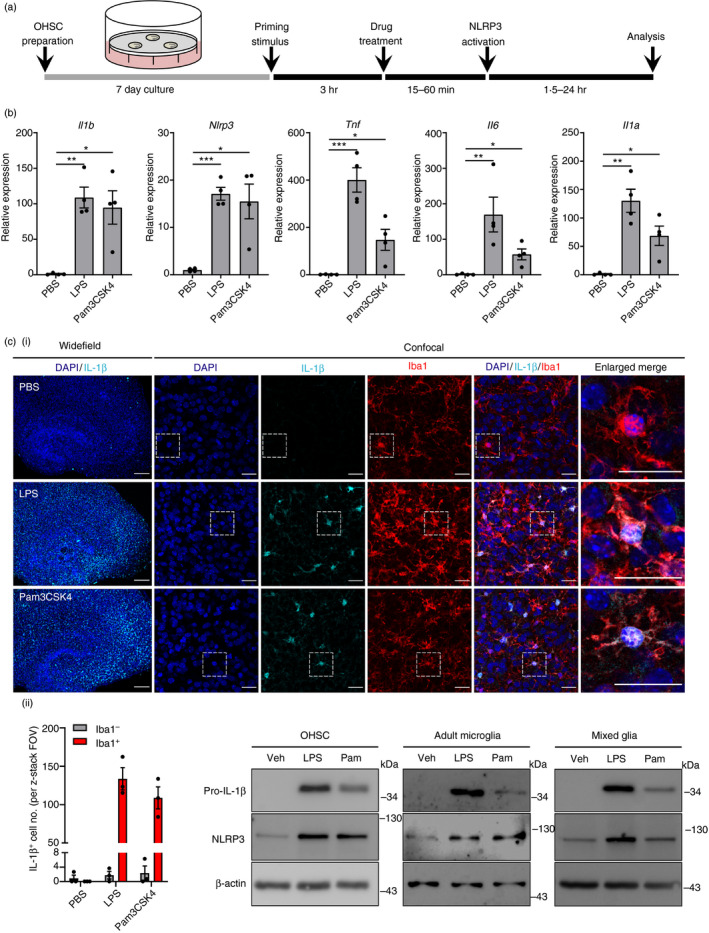
NACHT, LRR and PYD domain‐containing protein 3 (NLRP3) inflammasome priming by Toll‐like receptor (TLR) stimuli in organotypic hippocampal slice cultures (OHSCs). (a) Schematic of OHSC preparation and treatment protocol. (b–d) Wild‐type (WT) OHSCs were primed with vehicle (phosphate‐buffered saline; PBS), lipopolysaccharide (LPS) (1 µg/ml) or Pam3CSK4 *n* in culture medium containing serum. OHSCs were then processed for downstream RNA and protein analysis. (b) The expression of various inflammatory gene mRNA levels was assessed by quantitative PCR (*n* = 4). (ci) OHSCs were stained for nuclei (DAPI, blue), interleukin‐1*β* (IL‐1*β*) (cyan) and Iba1 (red) (*n* = 3). Images were acquired using wide‐field (5×) and confocal (63×) microscopy. Enlarged merge images show regions denoted by the white box. Scale bars are 200 µm (wide‐field) or 25 µm (confocal). (See also Supplementary material, Video S1). (cii) IL‐1*β*‐positive cells were quantified and assessed for co‐expression of Iba1 signal. (d) WT OHSCs, adult microglia and mixed glia lysates were assessed for pro‐IL‐1*β* and NLRP3 content by Western blotting (*n* = 3). Data are presented as mean ± SEM. Data were analysed using repeated‐measures one‐way analysis of variance with Dunnett’s *post‐hoc* test. ns, Not significant; *P < 0·05; **P < 0·01; ***P < 0·001.

A schematic demonstrating OHSC preparation and the treatment protocol is shown in Fig. 1(a). Canonical NLRP3 inflammasome‐dependent IL‐1*β* processing and release has a well‐defined two‐step activation mechanism. Priming is achieved *in vitro* through the activation of TLR4 or TLR2, both of which are expressed by microglia in the brain.^38^, ^39^ Consistent with this, treatment of WT OHSCs with the TLR4 agonist LPS, or the TLR2 agonist Pam3CSK4, induced the up‐regulation of *Il1b*, *Nlrp3*, *Tnf*, *Il6* and *Il1a* gene mRNA levels (Fig. 1b). *Pycard* (ASC), *Casp1*, *Gsdmd* and *Il18* were not significantly up‐regulated by either stimulus and were constitutively expressed (see Supplementary material, Fig. S1). Using immunofluorescence staining, pro‐IL‐1*β* protein production was observed throughout the OHSCs in response to LPS and Pam3CSK4 priming, and was expressed almost exclusively by Iba1‐positive microglia (Fig. 1ci, cii; and see Supplementary material, Video S1) ^40^. Up‐regulation of pro‐IL‐1*β* and NLRP3 protein expression was confirmed by Western blotting of OHSC lysates, as well as lysates of cultured adult murine microglia and primary murine mixed glia, which were run in parallel as a control (Fig. 1d).

### ASC speck formation can be visualized using ASC–citrine OHSCs

**Figure 2:**
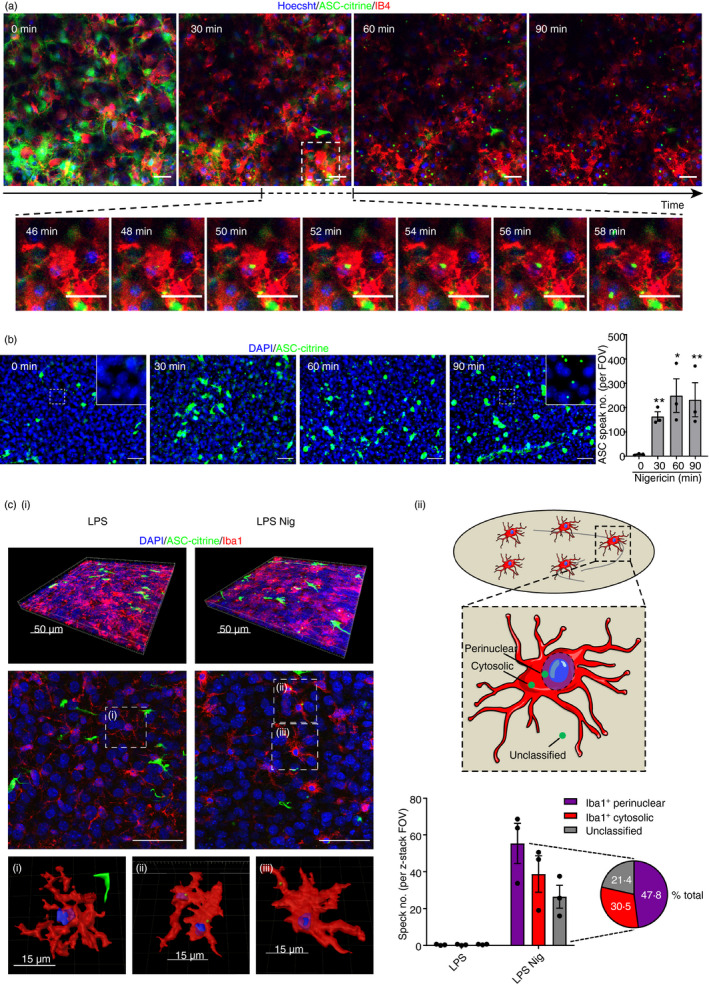
ASC speck formation can be visualized using ASC–citrine organotypic hippocampal slice cultures (OHSCs). (a) ASC–citrine OHSCs (at days 3–7 in culture) were lipopolysaccharide (LPS) primed (1 µg/ml, 3 hr) and medium was then replaced with phenol red‐free, serum‐free medium containing Hoechst (blue; 2 µg/ml) and isolectin GS‐IB4 AlexaFluor 594 conjugate (IB4; red; 5 µg/ml) and incubated for 2 hr. OHSCs were subsequently covered with 1·5 ml phenol red‐free, serum‐free media and then nigericin (10 µm) was spiked into the culture medium underneath the insert. The OHSCs were then placed into a confocal microscope chamber heated to 37° and imaged for 60–90 min. Image acquisition began 10 min after nigericin addition. The ASC–citrine signal is shown in green. Maximum intensity projection images are shown at 30‐min intervals after starting image acquisition. Enlarged region shows ASC speck formation at 2‐min intervals. Scale bars are 20 µm. The full video is shown in the Supplementary material (Video S2). (b and c) ASC–citrine OHSCs were LPS primed (1 µg/ml, 3 hr) before addition of nigericin (10 μm, 0–90 min). OHSCs were fixed and stained for nuclei (DAPI, blue). The ASC–citrine signal is shown in green. (b) Images were acquired using wide‐field microscopy at 20× magnification (*n* = 3). Scale bars are 50 μm. ASC speck formation was quantified. (ci) Images were acquired using confocal microscopy at 63× magnification (*n* = 3). Three dimensional (3D) z‐project fluorescence images are shown of representative OHSCs, along with a single z‐plane of each and 3D reconstructions of (i) an LPS‐primed cell, or cells containing (ii) perinuclear or (iii) cytosolic ASC specks. Scale bars are 50 µm where not indicated. (cii) A schematic of ASC speck spatial localization parameters is shown, indicating possible ASC speck locations. Specks were defined as perinuclear (≤ 2 μm from the nucleus), cytosolic (> 2 μm from the nucleus) or unclassified (did not colocalize with Iba1 signal). ASC specks were quantified. Data are presented as mean ± SEM. Data were analysed using repeated‐measures one‐way analysis of variance with Dunnett’s *post‐hoc* test. *P < 0·05; **P < 0·01.

Having confirmed that OHSCs had a normal NLRP3 inflammasome priming response, we next sought to investigate whether canonical NLRP3 inflammasome activation could also be elicited in OHSCs. To do this, we used a transgenic mouse in which fluorescent ASC–citrine fusion protein is expressed.^36^ The response of ASC–citrine OHSCs to priming stimuli was similar to WT OHSCs (Fig. 2a,b). Subsequently, ASC–citrine OHSCs were primed with LPS and incubated in isolectin GS‐IB4 AlexaFluor 594 conjugate (IB4), which primarily stains microglia in the central nervous system, before treatment with the NLRP3 activator nigericin.^41^ ASC speck formation was assessed using time‐lapse confocal microscopy, and image acquisition was begun 10–20 min after nigericin addition (see Supplementary material, Video S2). Images at 30‐min time‐points after starting image acquisition are shown, as well as an enlarged region of the image at 2‐min intervals (Fig. 2a). ASC specks formed throughout the time–course of nigericin treatment, and these ASC specks were predominantly in IB4‐positive microglia (Fig. 2a). In some cases, ASC specks had already formed before acquisition of the first image. The ASC specks formed in response to nigericin remained cell‐associated for the duration of the recording in the absence of a caspase‐1 inhibitor, suggesting that they were not immediately released into the extracellular environment as a result of cell death (Fig. 2a). These findings were validated by using wide‐field microscopy to assess ASC speck formation in ASC–citrine OHSCs that had been fixed at 30‐min time‐points after nigericin treatment. Similar to the live imaging, ASC speck formation was detected in ASC–citrine OHSCs after 30 min of nigericin treatment, with the number of ASC specks increasing up to 60 min following treatment (Fig. 2b). It is important to note that large aggregates of ASC–citrine were observed in all ASC–citrine OHSCs, even in the absence of treatment, possibly resulting from the overexpression of ASC, which has a high propensity to aggregate because of its prion‐like properties (Fig. 2b; and see Supplementary material, Fig. S2C).^36^, ^42^ These aggregates were much larger than ASC specks and were excluded from ASC speck quantification.

The characteristics of ASC speck formation in OHSCs, including the cell types in which they formed and their subcellular localization, were further investigated. Representative 3D projections and z‐planes of ASC speck‐expressing OHSCs are shown (Fig. 2ci). 3D reconstructions illustrate the peri‐nuclear and peripheral subcellular locations of the ASC speck. Approximately 80% of ASC specks formed in Iba1‐positive microglia, with 50% of total ASC specks observed in the perinuclear region of Iba1‐positive microglia and 30% present in the peripheral cytosol (Fig. 2cii). The remaining ASC specks could not be classified. They did not appear to be present in Iba1‐positive microglia but could be extracellular or in non‐labelled cells (Fig. 2cii).^43^

### Validation of ASC speck formation in WT OSHCs

**Figure 3:**
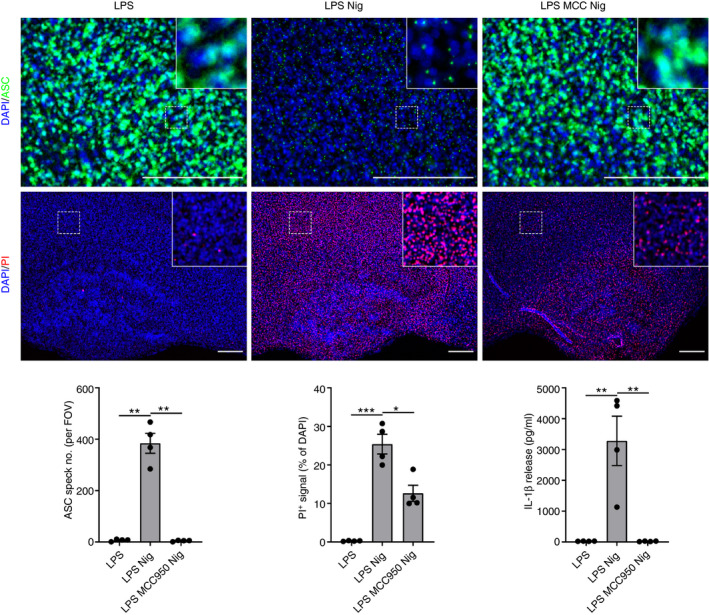
Validation of ASC speck formation in wild‐type organotypic hippocampal slice cultures (WT OSHCs). WT OHSCs were primed with lipopolysaccharide (LPS) (1 µg/ml, 3 hr) before treatment with MCC950 (MCC; 10 μm, 15 min) where appropriate and addition of nigericin (10 μm, 0–90 min; *n* = 4). Propidium iodide (PI; red, 25 µg/ml) was added for the final 30 min of nigericin treatment. OHSCs were probed for nuclei (DAPI, blue) and ASC (green) by immunofluorescence staining. Images were acquired using wide‐field microscopy at 20× (ASC) and 5× (PI) magnification. Scale bars are 200 μm. The ASC speck number was quantified. The area of PI‐positive staining is expressed as a % of total area of DAPI staining. Supernatant was also assessed for IL‐1*β* content by ELISA. Data are presented as mean ± SEM. Data were analysed using repeated‐measures one‐way analysis of variance with Dunnett’s *post‐hoc* test. ns, Not significant; *P < 0·05; **P < 0·01; ***P < 0·001.

NLRP3‐dependent ASC speck formation was next investigated in WT OHSCs expressing only endogenous ASC. WT OHSCs were treated with LPS followed by nigericin and were then fixed and probed for ASC using immunofluorescence staining as described above. Diffuse ASC staining was observed in LPS‐primed OHSCs, whereas ASC specks could be detected in LPS‐primed OHSCs treated with nigericin (Fig. 3). Interleukin‐1*β* was also detected in the culture medium, indicating that these ASC specks were active (Fig. 3). The NLRP3 inflammasome inhibitor MCC950^44^ blocked both ASC speck formation and IL‐1*β* release, confirming that these processes were NLRP3 dependent (Fig. 3). Inflammasome activation also induces pyroptotic cell death, and to measure this, OHSCs were incubated with PI, added for the final 30 min of nigericin treatment ^23^. Nigericin treatment induced cell death throughout the OHSCs, and this was reduced by MCC950, suggesting that there was inflammasome‐dependent pyroptotic death in response to nigericin (Fig. 3).

### Different canonical NLRP3 stimuli induce inflammasome activation

**Figure 4:**
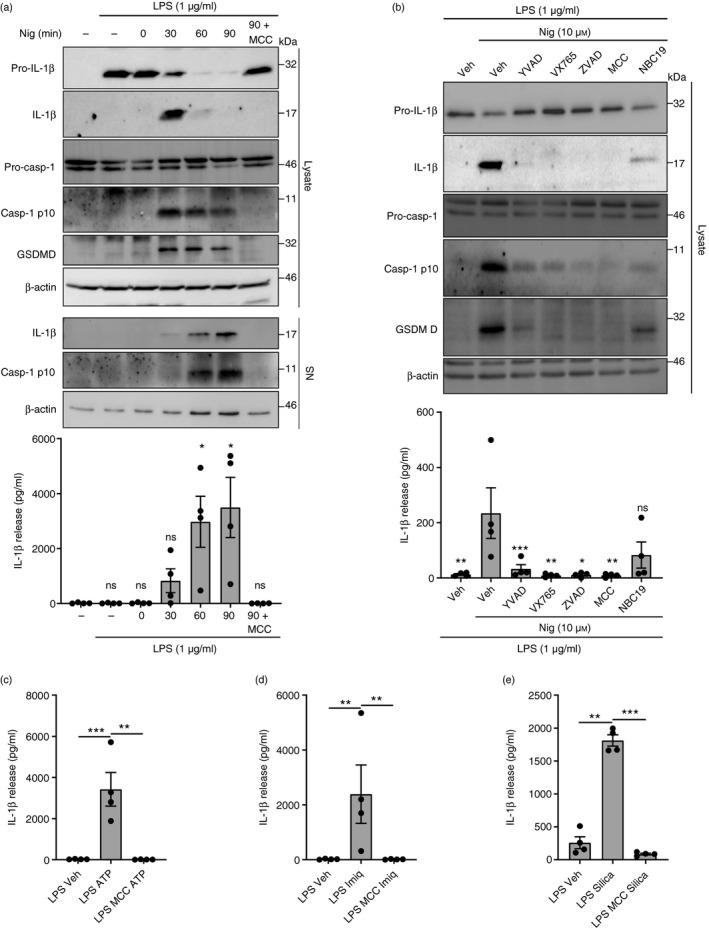
Different canonical NACHT, LRR and PYD domain‐containing protein 3 (NLRP3) stimuli induce inflammasome activation. (a) Wild‐type organotypic hippocampal slice cultures (WT OHSCs) were untreated or primed with lipopolysaccharide (LPS) (1 μg/ml, 3 hr), before treatment with MCC950 (10 μm, 15 min), before the addition of nigericin (10 μm, 0–90 min; *n* = 4). OHSC lysates and supernatants were probed by Western blotting for markers of inflammasome activation. Supernatants were also assessed for interleukin‐1*β* (IL‐1*β*) content by ELISA. (b) WT OHSCs were primed with LPS (1 μg/ml, 3 hr), before treatment with ac‐YVAD‐cmk (100 μm, 15 min), VX‐765 (10 μm, 15 min), ZVAD‐fmk (50 μm, 15 min), MCC950 (10 μm, 15 min) or NBC19 (25 μm, 15 min), before the addition of nigericin (10 μm, 30 min; *n* = 4). OHSC lysates were probed for markers of inflammasome activation by Western blotting. Interleukin‐1*β* release into the supernatant was measured by ELISA. (c–e) WT OHSCs were primed with LPS (1 µg/ml, 3 hr) before treatment with MCC950 (10 μm, 15 min), before the addition of (c) ATP (5 mm, 90 min; *n* = 4), (d) imiquimod (75 μm, 2 hr; *n* = 4) or (e) silica (3 μl, 3 mg/ml, 24 hr; *n* = 4). Silica was pipetted directly on top of each OHSC. Supernatant was assessed for IL‐1*β* content by ELISA. Data are presented as mean ± SEM. Data were analysed using repeated‐measures one‐way analysis of variance with Dunnett’s *post‐hoc* test. ns, Not significant; *P < 0·05; **P < 0·01; ***P < 0·001.

To further characterize canonical NLRP3 inflammasome responses, LPS‐primed WT OHSCs were treated with nigericin for 90 min, and Western blotting was used to measure markers of inflammasome activation. Active caspase‐1 p10, cleaved gasdermin D and mature IL‐1*β* were detected in the OHSCs as early as 30 min after nigericin treatment, with less mature IL‐1*β* and caspase‐1 p10 detected in the OHSCs at the 60‐ and 90‐min time‐points (Fig. 4a). Western blotting and ELISA of the culture medium confirmed that IL‐1*β* and caspase‐1 were being released from the OHSCs after their cleavage (Fig. 4a). MCC950 blocked caspase‐1 and IL‐1*β* activation and release, as well as gasdermin D cleavage (Fig. 4a). Caspase‐1 inhibition with Ac‐YVAD‐cmk or VX‐765,^45^ pan‐caspase inhibition with ZVAD‐fmk, or NLRP3 inflammasome inhibition using MCC950 or the recently described NBC19^46^ all inhibited caspase‐1 activation, gasdermin D cleavage, and IL‐1*β* processing and release from WT OHSCs after 30 min of nigericin treatment (Fig. 4b).

A range of stimuli has been described to activate the canonical NLRP3 pathway, including extracellular ATP, imiquimod and particulates such as silica crystals.^13^, ^14^, ^15^, ^17^ Hence, additional NLRP3 agonists were tested to determine their potential to activate the NLRP3 inflammasome in OHSCs. LPS‐primed WT OHSCs were treated with ATP, imiquimod or silica, and these induced caspase‐1 activation, gasdermin D cleavage and release of IL‐1*β*, all of which were prevented by MCC950 treatment (Fig. 4c–e; see Supplementary material, Fig. S3A–C). Mature IL‐1*β* and caspase‐1 p10 were also detected in the culture medium by Western blotting, although caspase‐1 p10 was often present at low levels (Fig. S3A–C). ASC speck formation and IL‐1*β* release in response to ATP and imiquimod were further confirmed in ASC–citrine OHSCs (Fig. S3D,E). We then compared the NLRP3 inflammasome response of OHSCs with other microglial systems. Interleukin‐1*β* release and cell death in response to nigericin, ATP, imiquimod and silica were consistent in both primary adult microglia and primary mixed glial cultures, and IL‐1*β* release was inhibited by MCC950 in all cases (Fig. S4A–H).

## Discussion

Microglial NLRP3 inflammasome responses play an important role in neuroinflammation associated with brain pathology.^2^, ^5^ However, a gap exists between *in vivo* observations of microglial neuroinflammatory mechanisms and respective *in vitro* models, which currently focus mainly on pure microglial monolayer cultures.^34^, ^47^ This is reflected by recent identification of heterogeneous microglial populations within the central nervous system by single‐cell RNA‐Seq, highlighting the need for more complex, integrated microglial models to better reflect their physiology.^48^, ^49^, ^50^ To address this, we investigated microglial NLRP3 inflammasome responses in a complex 3D *ex vivo* brain‐like environment. We demonstrated that OHSCs exhibit robust microglial NLRP3 inflammasome priming and activation, indicating that OHSCs can aid the generation of important new insights into microglial neuroinflammatory mechanisms.

The OHSC model of NLRP3 inflammasome activation in microglia begins to bridge the gap between mechanistic insights into NLRP3 regulation derived from *in vitro* studies and *in vivo* observations that demonstrate NLRP3 inflammasome relevance in the brain.^31^, ^32^, ^33^, ^51^ Our findings are supported by reports of IL‐1*β* release from murine OHSCs and acutely isolated rat hippocampal slices in response to LPS and ATP treatment.^29^, ^30^, ^52^ Spontaneous epileptiform activity has also been suggested to increase NLRP3 and inflammatory cytokine expression in rat OHSCs.^53^ Hence, by using OHSCs, we can begin to dissect relevant pathophysiological mechanisms of neuroinflammation in an environment more representative of the brain parenchyma.

Although there are several limitations to the use of OHSCs, they were selected primarily because they maintain a 3D cellular architecture, preserving the interactions and connectivity between microglia, astrocytes and neurons. These cell–cell interactions are critical for microglial function, as astrocytes secrete various factors such as transforming growth factor‐*β*_2_, colony‐stimulating factor‐1 and cholesterol that promote microglial survival.^54^ Furthermore, the OHSC vasculature is still present during culture, despite the absence of blood flow.^55^, ^56^ Other studies have used acutely prepared hippocampal slices from adult rodents to investigate microglial function.^30^, ^57^ Although these models confer many benefits for studying microglia *ex vivo*, there are major differences with OHSCs. For example, microglia from acute brain slices develop an amoeboid morphology in response to neuronal injury during the 24‐hr period after sectioning.^58^ Any resulting investigations would therefore be performed in an already inflammatory environment. However, in OHSCs, the inflammatory response induced by the initial injury subsides over the course of the culture, meaning that by the time of inflammatory perturbation, the microglia appear more ramified and the production of inflammatory cytokines is reduced.^59^ Our data support this, as the expression of various cytokines was negligible after 7 days of culture. The study of OHSCs repopulated with adult microglia has also been characterized.^60^ Various methods have employed human induced pluripotent stem cells, or microglia acquired from post‐mortem tissue, to study the role of human microglia.^61^, ^62^ However, in isolation, these models lack the complex interplay between microglia, astrocytes and neurons. 3D cerebral organoids can be developed from human induced pluripotent stem cells to investigate human microglia in a more brain‐like environment, but they lack vasculature and typically exhibit high variability among individual organoids, although recent progress has been made in this regard.^63^, ^64^, ^65^

We have demonstrated various fundamental applications of OHSCs with regard to studying microglial inflammatory responses, presenting opportunities to further interrogate neuroinflammation. We highlight OHSCs as a model in which to investigate mechanisms of NLRP3 inflammasome priming in microglia. For example, the precise mechanisms of microglial priming in acute brain injuries such as stroke, including identification of the endogenous damage‐associated stimuli, are unclear. Furthermore, we show that live imaging can be performed on OHSCs, offering a tractable system for easier investigation and hypothesis testing than *in vivo* imaging, but one that maintains physiological relevance compared with purely *in vitro* studies. Implications of NLRP3 inflammasome activation could be studied in OHSCs, such as characterizing the longer‐term fate of ASC specks following pyroptotic cell death. Furthermore, because of the absence of blood and lymphatic flow, intrinsic properties of the OHSCs can be isolated. Hence, it can be determined whether ASC speck clearance can be mediated by the cells within the tissue itself, such as by phagocytosis, or whether cellular infiltration may be a requirement for the resolution of an inflammatory response. Exogenous addition of peripheral cells to OHSCs has previously been performed, and this would allow examination of the effects of infiltrating peripheral cells.^66^ Heterogeneity of inflammatory responses could also be investigated in slice cultures prepared from different brain regions, as well as between male and female mice.

We have demonstrated that OHSCs are a tractable model to interrogate microglial NLRP3 inflammasome responses. Our data indicate that, in OHSCs, microglia are NLRP3 inflammasome‐competent cells that exhibit robust canonical NLRP3 inflammasome priming and activation. This model provides a platform from which to address a wide range of critical questions in the field. Incorporating OHSCs into the existing pool of established cell and tissue models for studying inflammasomes would help to advance the study of brain inflammasome responses, further enhancing our understanding of the role of inflammasomes and microglia in brain inflammation and disease.

## Author contributions

Conceptualization, E.L., D.B. and S.M.A; methodology, C.H., E.L. and D.B.; investigation, C.H., E.L., E.R‐C. and J.C.; resources, T‐C.T.; writing – original draft, C.H.; writing – review and editing, C.H., E.L., D.B. and S.M.A; supervision, D.B. and S.M.A.; funding acquisition, D.B. and S.M.A.

## Acknowledgements

The ASC–citrine mice were a kind gift from Douglas Golenbock (University of Massachusetts Medical School).

## Funding information

This work was funded by the MRC (Grant No. MR/N003586/1 to D.B. and S.M.A.) and was also funded by an MRC PhD studentship to C.H. (Grant No. MR/N013751/1). The Bioimaging Facility microscopes used in this study were purchased with grants from BBSRC, Wellcome and the University of Manchester Strategic Fund.

## Disclosures

The authors declare no conflicts of interest.

## Supporting information


**Video S1.** Interleukin‐1*β* production in response to lipopolysaccharide priming.

**Video S2.** Time‐lapse imaging of ASC speck formation in ASC–citrine organotypic hippocampal slice cultures.

**Figure S1.** NLRP3 inflammasome priming by Toll‐like receptor stimuli in organotypic hippocampal slice cultures.
**Figure S2.** NLRP3 inflammasome priming is consistent in ASC–citrine organotypic hippocampal slice cultures, but they exhibit large aggregates.
**Figure S3.** A range of damage‐associated molecular patterns can activate the canonical NLRP3 inflammasome in ASC–citrine organotypic hippocampal slice cultures.
**Figure S4.** Canonical NLRP3 inflammasome activation in microglial cultures.

## Data Availability

The data that support the findings of this study are available from the corresponding author upon reasonable request.
