# Supplementary material for: Hallmarks of NLRP3 inflammasome activation are observed in organotypic hippocampal slice culture
Source: Immunology. 2020 Jun 22;161(1):39–52. doi: 10.1111/imm.13221 (PMC7450173; doi:10.1111/imm.13221)
Supplement: Supplementary file 3 — Figure S1. NLRP3 inflammasome priming by Toll‐like receptor stimuli in organotypic hippocampal slice cultures. Figure S2. NLRP3 inflammasome priming is consistent in ASC–citrine organotypic hippocampal slice cultures, but they exhibit large aggregates. Figure S3. A range of damage‐associated molecular patterns can activate the canonical NLRP3 inflammasome in ASC–citrine organotypic hippocampal slice cultures. Figure S4. Canonical NLRP3 inflammasome activation in microglial cultures. [file IMM-161-39-s003.docx]

**
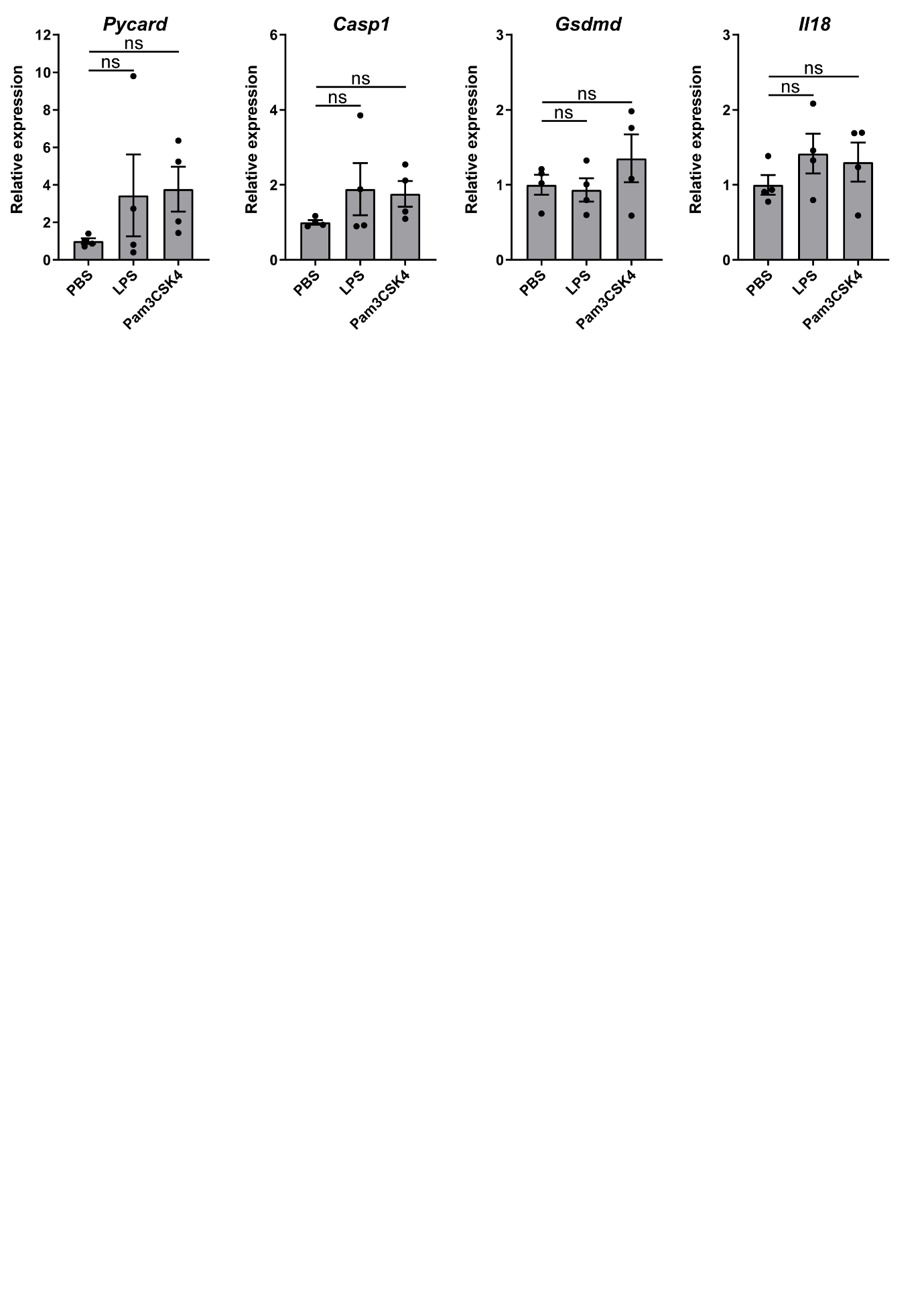
**

**Figure S1. NLRP3 inflammasome priming by TLR stimuli in OHSCs.** WT OHSCs were primed with vehicle (PBS), LPS (1 µg ml^–1^) or Pam3CSK4 (100 ng ml^–1^, 3 h) in culture medium containing serum. The expression of various inflammatory gene mRNA levels was assessed by qPCR (n=4). Data are presented as mean ± SEM. Data were analysed using repeated-measures one-way ANOVA with Dunnett’s post-hoc test. ns, Not significant.

**
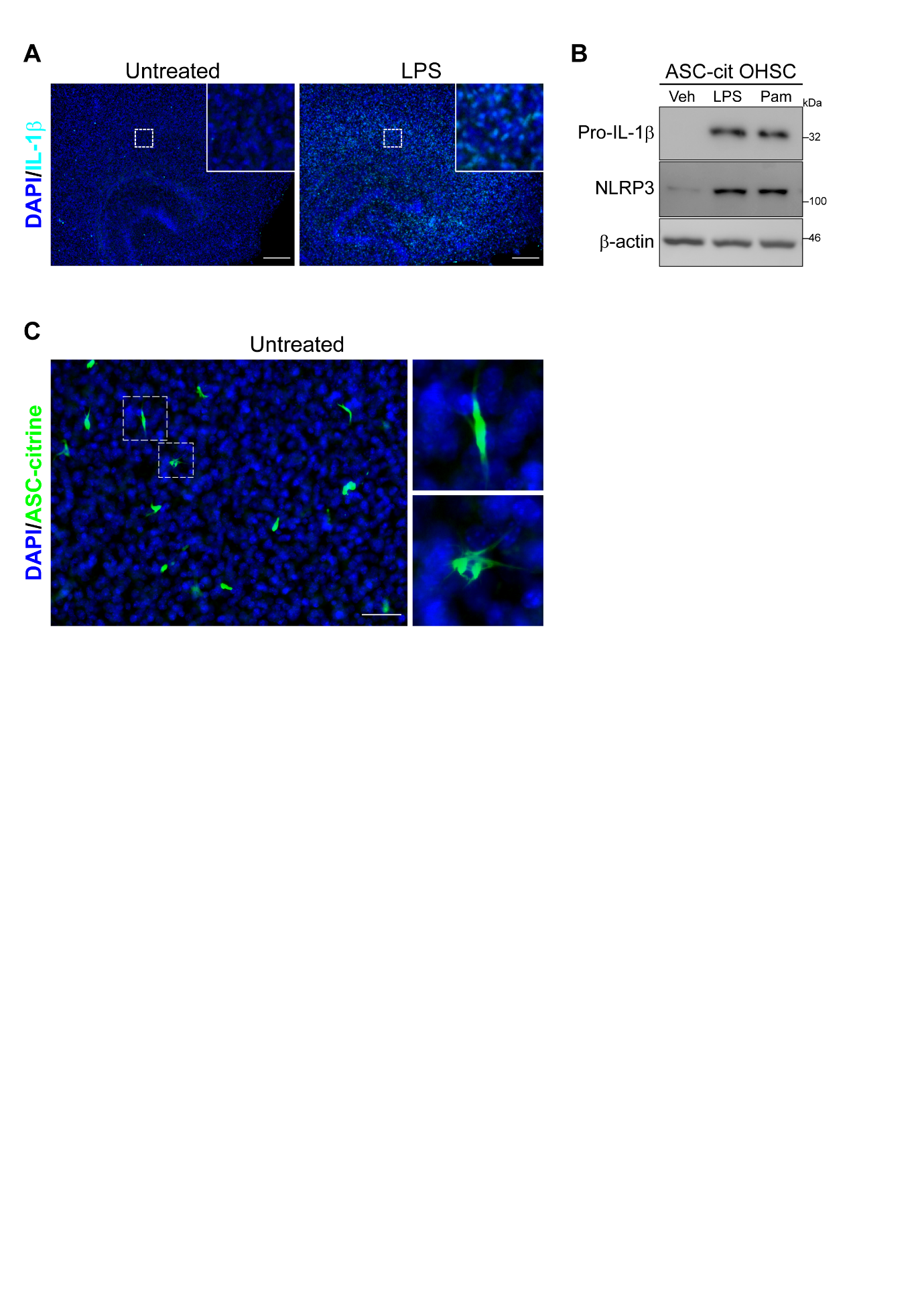
**

**Figure S2. NLRP3 inflammasome priming is consistent in ASC–citrine OHSCs, but they exhibit large aggregates.** (**A**) ASC–citrine OHSCs were left untreated or primed with LPS (1 µg ml^–1^, 3 h) in culture medium containing serum (n=3). OHSCs were then fixed and stained for nuclei (DAPI, blue) or IL-1β (cyan). Representative images are shown, excluding the ASC–citrine signal. Images were captured using a fluorescent widefield microscope at 5X magnification. Scale bars are 200 µm. (**B**) ASC–citrine OHSCs were primed with vehicle (PBS), LPS (1 µg ml^–1^) or Pam3CSK4 (100 ng ml^–1^, 3 h; n=3). OHSC lysates were then probed for pro-IL-1β and NLRP3 content by western blotting. (**C**) Untreated ASC–citrine OHSCs were fixed and stained for nuclei (DAPI, blue). The ASC–citrine signal is shown in green. The image was captured using a fluorescent widefield microscope at 20X magnification. Scale bar is 50 µm.

**
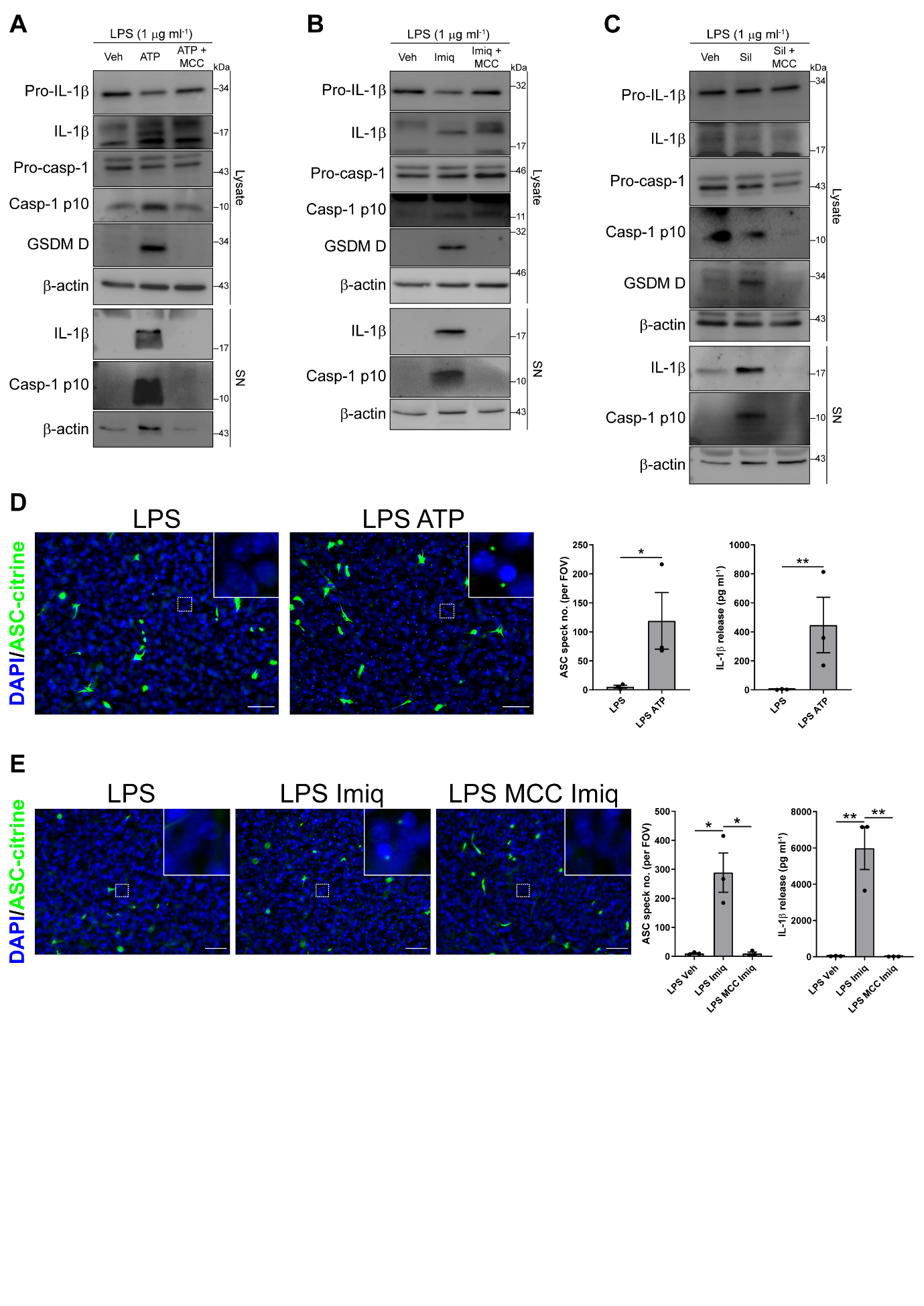
**

**Figure S3. A range of DAMPs can activate the canonical NLRP3 inflammasome in ASC–citrine OHSCs.** (**A**–**C**) WT OHSCs were primed with LPS (1 µg ml^–1^, 3 h) prior to treatment with MCC950 (MCC; 10 μM, 15 min) where appropriate and addition of (**A**) ATP (5 mM, 90 min; n=4), (**B**) imiquimod (75 μM, 2 h; n=4) or (**C**) silica (3 µl, 3 mg ml^–1^, 24 h; n=4). OHSC lysate and supernatants were probed by western blotting for markers of inflammasome activation. Measurement of IL‑1β release is shown in Figure 3D–F. (**D**–**E**) ASC–citrine OHSCs were primed with LPS (1 µg ml^–1^, 3 h) prior to treatment with MCC950 (MCC; 10 μM, 15 min) where appropriate and the addition of (**D**) ATP (5 mM, 90 min; n=3) or (**E**) imiquimod (75 μM, 2 h; n=3). OHSCs were fixed and stained for nuclei (DAPI, blue). The ASC–citrine signal is shown in green. Images were acquired using widefield microscopy at 20X magnification. Scale bars are 50 μm. The total ASC speck number was quantified. The supernatant was assessed for IL-1β content by ELISA. Data are presented as mean ± SEM. Data were analysed using an unpaired *t*-test or repeated-measures one-way ANOVA with Dunnett’s *post-hoc* test. **P*<0.05; ***P*<0.01.


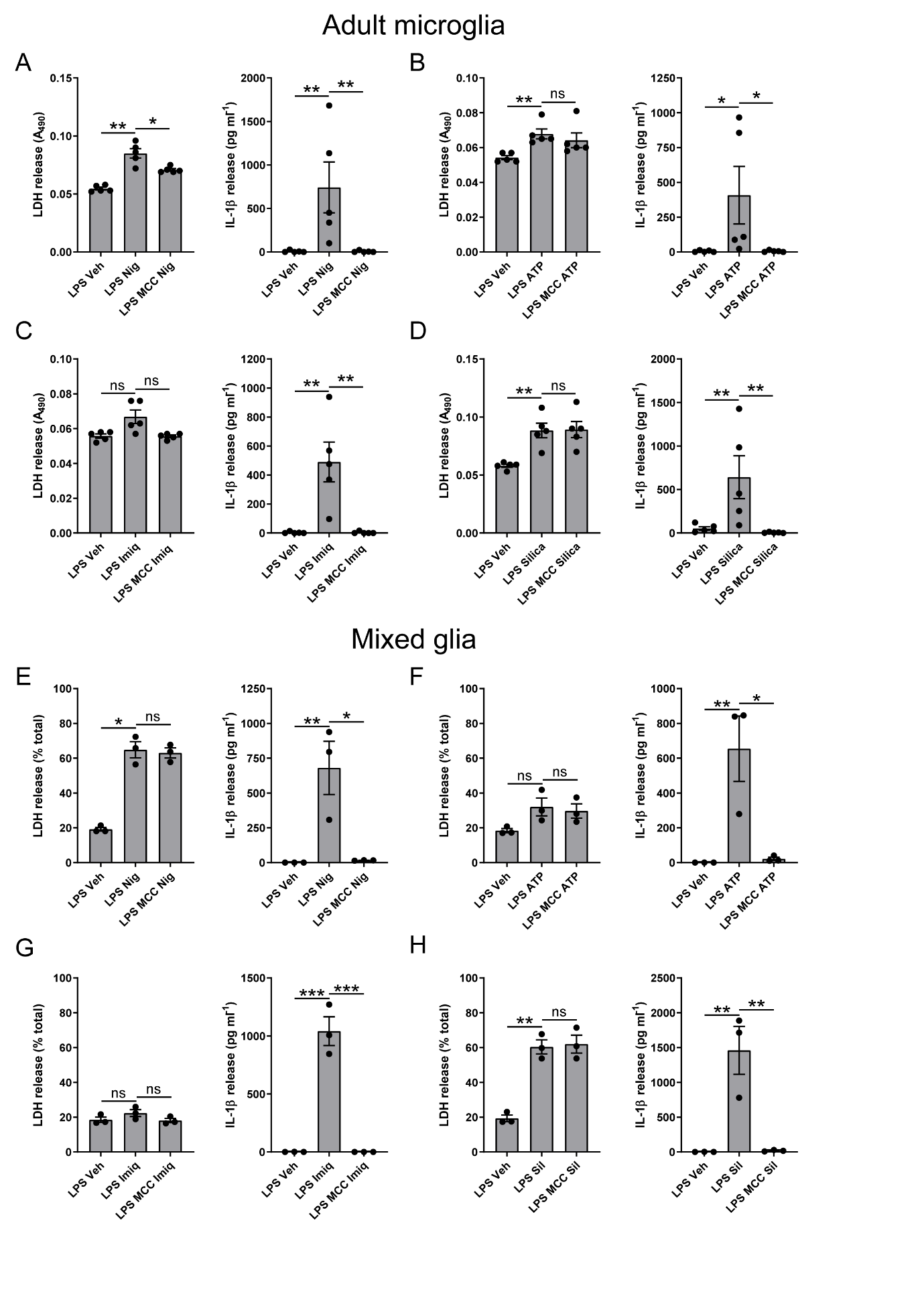


**Figure S4. Canonical NLRP3 inflammasome activation in microglial cultures.** (**A**–**D**) Adult microglia were primed with LPS (1 µg ml^–1^, 3 h) prior to treatment with MCC950 (MCC; 10 μM, 15 min) and addition of (**A**) nigericin (10 μM, 60 min; n=5), (**B**) ATP (5 mM, 60 min; n=5), (**C**) imiquimod (75 µM, 2 h; n=5) or (**D**) silica (300 μg ml^–1^, 24 h; n=5). Supernatants were assessed for LDH release (cell death) and IL-1β content by ELISA. (**E**–**H**) Mixed glia were primed with LPS (1 µg ml^–1^, 3 h) prior to treatment with MCC950 (10 μM, 15 min) and addition of (**E**) nigericin (10 μM, 60 min; n=3), (**F**) ATP (5 mM, 60 min; n=3), (**G**) imiquimod (75 µM, 2 h; n=3) or (**H**) silica (300 μg ml^–1^, 4 h; n=3). Supernatants were assessed for LDH release (cell death) and IL-1β content by ELISA. Data are presented as mean ± SEM. Data were analysed using repeated-measures one-way ANOVA with Dunnett’s *post-hoc* test. ns, Not significant; **P*<0.05; ***P*<0.01; ****P*<0.001.

**Video S1. IL-1β production in response to LPS priming.** WT OHSCs were primed with LPS (1 µg ml^–1^, 3 h) in culture medium containing serum. OHSCs were stained for nuclei (DAPI, blue), IL-1β (cyan) and Iba1 (red) (n=3). Images were acquired using confocal (63X) microscopy. Scale bar is 25 µm. Z-plane distance between sequential images is 0.5 µm. See also Figure 1C.

**Video S2. Time-lapse imaging of ASC speck formation in ASC–citrine OHSCs.** ASC–citrine OHSCs were LPS primed (1 µg ml^–1^, 3 h) and then the medium was replaced with phenol red-free, serum-free medium containing Hoechst (blue; 2 µg ml^–1^) and isolectin GS-IB4–Alexa Fluor^™^ 594 conjugate (IB4; red; 5 µg ml^–1^) and incubated for 2 hours. OHSCs were subsequently covered with 1.5 ml phenol red-free, serum-free medium, and then nigericin (10 µM) was spiked into the culture medium underneath the insert. The OHSCs were then placed into a confocal microscope chamber heated to 37°C and imaged for 60–90 minutes. The ASC–citrine signal is shown in green. Scale bar is 50 μm. See Figure 2A for maximum projection images at 30-minute intervals.
